# Investigation of gut microbiota in pediatric patients with peanut allergy in outpatient settings

**DOI:** 10.3389/fped.2025.1509275

**Published:** 2025-02-12

**Authors:** Shouming Li, Jingyi Huang, Yunyun Xie, Di Wang, Xin Tan, Yufan Wang

**Affiliations:** ^1^ Department of Child Health Care, Jiangxi Children’s Hospital, Nanchang, China; ^2^ School of Pediatrics, Medical College of Nanchang University, Nanchang, China

**Keywords:** peanut allergy, gut microbiota, 16S rRNA, food allergy, children

## Abstract

**Objective:**

Investigate the diversity of the gut microbiota in children with peanut allergies and assess its association with allergic reactions. Identify potential gut microbial biomarkers for the diagnosis and treatment of peanut allergies.

**Methods:**

Twenty-nine children with peanut allergy who visited the hospital from December 2020 to December 2022 were selected as the test group (PA), and twenty-seven healthy children who underwent physical examination during the same period and tested negative for peanut IgE were selected as the control group (Ctl). The differences in gut microbiota between the two groups were compared. The study enrolled 29 children with peanut allergy (PA group) and 27 healthy children (Ctl group) from December 2020 to December 2022. The PA group was defined by a positive reaction to peanut-specific IgE tests, while the Ctl group tested negative for peanut IgE and had no history of allergies. Fecal samples were collected and genomic DNA was extracted for 16S rRNA gene sequencing to assess gut microbiota composition. Alpha diversity indices, including the sob, ace, chao, shannon, and simpson indices, were calculated to assess microbial community richness and diversity. Beta diversity was analyzed using Principal Coordinate Analysis (PCoA) and Partial Least Squares Discriminant Analysis (PLS-DA) to compare microbial community structures between the PA and Ctl groups.

**Results:**

The study identified significant differences in gut microbiota diversity between children with peanut allergy (PA group) and healthy controls (Ctl group). The PA group exhibited reduced alpha diversity, indicated by lower sob, ace, and chao indices (FDR ≤ 0.05), and a significantly lower Shannon index (FDR ≤ 0.01). Beta diversity analysis revealed distinct microbial community structures between the two groups. Notably, the PA group showed an increase in Proteobacteria and a decrease in Firmicutes and Bacteroidetes, with significant changes at the genus level, including lower relative abundance of Bacteroides and Faecalibacterium, and higher relative abundance of Bifidobacterium and Lactobacillus (FDR ≤ 0.05 or FDR ≤ 0.01). Correlation analysis highlighted a strong negative correlation between IgE levels and specific microbial groups, such as Alistipes and CAG-352 (FDR ≤ 0.001), and a positive correlation with Veillonella. Blood routine indicators were also found to be correlated with gut microbiota composition.

**Conclusion:**

The findings of this study provide compelling evidence that gut microbiota diversity and composition are significantly altered in children with peanut allergy. The observed shifts in microbial communities, particularly the increase in Proteobacteria and the decrease in beneficial bacteria such as Firmicutes and Bacteroidetes, underscore the potential role of the gut microbiome in the pathogenesis of peanut allergy. These results suggest that modulating the gut microbiota may be a viable therapeutic strategy for managing peanut allergy and highlight the need for further research to explore the clinical implications of these microbial changes.

## Introduction

Allergic reactions are a common health issue in children, with their incidence rates showing an increasing trend worldwide. This study adheres to the definition of allergy by the National Institute of Allergy and Infectious Diseases (NIAID) and the World Health Organization (WHO), which states that “allergy is an abnormal immune response to usually harmless foreign substances (allergens)” (1, 2). Specifically, our research focuses on IgE-mediated allergic reactions, which typically manifest symptoms rapidly upon exposure to allergens, including but not limited to skin itching, wheezing, and gastrointestinal symptoms. As a common food allergen, peanut-induced allergic reactions can lead to long-term health issues in children, affecting their growth and development, and imposing economic and psychological burdens on families. Therefore, gaining a deep understanding of the mechanisms behind peanut allergy is of great significance for the prevention and treatment of such allergic reactions.

Peanuts have high nutritional value, containing abundant fats and proteins, as well as various minerals, vitamins, and other nutrients (3–5). However, they are also one of the most important food allergens (6–8). Peanut allergy reactions are mediated by type I hypersensitivity reactions, which are induced by mast cells under the action of peanut allergens. The main allergens in peanuts are primarily found in the skin and interior of the peanut. Arah1 and Arah3 are mainly found in the skin, while Arah2, Arah6, and Arah8 are mainly found in the interior (9). These allergens are difficult to completely remove during peanut processing, so even after processing, peanuts can still cause allergic reactions (10). The incidence of peanut allergy reactions is long-term and severe, and it is on the rise, a trend that has affected the development and utilization of peanuts. As one of the allergenic foods, consuming peanuts or products containing peanuts can affect the normal growth and development of children, exacerbating the economic and psychological burden on families (11). In addition, peanut allergens can cause serious allergic complications, such as asthma (12), refractory peptic ulcers (13), and allergic rhinitis (14). Research data released at the 2017 Annual Scientific Meeting of the American College of Allergy, Asthma, and Immunology (ACAAI) showed that the incidence of peanut allergy in children has increased by an alarming 21% since 2010 (15, 16), and has become the leading cause of food allergy-related deaths (17). With the development of economic globalization, the production, distribution, and consumption of food will become increasingly internationalized, and peanut allergy will become a globally shared concern. However, there are few clinical case reports of peanut allergy in China. The results of a survey of food allergy patients in Beijing showed that about 4% of allergy sufferers were allergic to peanuts (18), and in Urumqi, peanuts were the main food allergen (19), but the incidence rate is unknown. The primary cause of this discrepancy may stem from differences in how Chinese children consume peanuts compared to their Western counterparts, as well as the distinct methods of cooking peanuts in the East and West (20). Typically, roasting can heighten the allergenicity of certain foods, whereas boiling may diminish the allergenicity of peanuts. In Western cultures, peanuts are frequently consumed in roasted forms, while in China, parents are more likely to prepare peanuts for their children by boiling (21). Nevertheless, cases of peanut allergy in children still occur sporadically and may be related to the consumption of peanut butter, peanut oil, and roasted peanuts in the family. There is a certain correlation between gut dysbiosis and reduced microbial diversity and peanut allergy (22). Research has found that gut dysbiosis and decreased microbial diversity may increase the risk of individuals developing peanut allergies (23). Gut dysbiosis can lead to abnormal immune system reactions, increasing the likelihood of individuals having allergic reactions to peanut allergens. Additionally, gut dysbiosis may also impair the intestinal barrier function, making it easier for peanut allergens to enter the bloodstream and trigger allergic reaction. With the increasing prevalence of peanut allergy affecting a segment of the pediatric population, there is an urgent need to explore the potential contributing factors and mechanisms. To address this need, we have conducted high-throughput testing of fecal gut microbiota from cases of peanut allergy we have encountered in clinical practice. Through this study, we aim to:

1. Investigate the gut microbiota composition in children with peanut allergy and compare it with age-matched non-allergic control groups.
2. Assess the relationship between gut microbial diversity and the severity of peanut allergy reactions.

These objectives are designed to enhance our understanding of the role of gut microbiota in pediatric peanut allergy and to identify novel targets for therapeutic intervention.

## Materials and methods

### Study subjects

The study subjects are children diagnosed with peanut allergy at the outpatient department of Jiangxi Provincial Children's Hospital from December 2020 to December 2022, aged between 18 and 36 months and were classified as the PA group. Diagnostic criteria for peanut allergy: A history of exposure or ingestion of peanut butter, peanut oil obtained by pressing, and foods such as pies, cakes, and biscuits containing peanuts, followed by immediate symptoms including vomiting, stomach cramps, indigestion, diarrhea, difficulty breathing, throat swelling, a sudden drop in blood pressure, pale skin or bluish lips, syncope, and dizziness. A positive peanut-specific IgE test result upon presentation at the outpatient department. The inclusion criteria were: (1) aged 18–36 months; (2) met the diagnostic criteria for peanut allergy. These reactions lasted for 2–4 h. (3) all are breasted. The exclusion criteria were: (1) no symptoms related to peanut allergy; (2) use of antibiotics, systemic corticosteroids, probiotics, prebiotics, and other drugs during follow-up; (3) pet ownership; (4) acute or chronic diseases, such as infectious diseases, liver and kidney dysfunction, blood diseases, autoimmune diseases, congenital diseases, and genetic metabolic diseases.

Healthy children who underwent physical examinations during the same period were selected as the Ctl group. The inclusion criteria were: (1) no clinical symptoms related to allergies; (2) the exclusion criteria were: (1) either parent had a history of allergies; (2) use of antibiotics, systemic corticosteroids, probiotics, prebiotics, and other drugs during follow-up; (3) pet ownership; (4) poor compliance of parents.

Symptom Management: The children in our study were not identified through planned challenge tests for allergic reactions; instead, they were included in the study because they were exposed to peanuts in their daily lives and developed allergic symptoms. Our research design was aimed at simulating real-world allergic reaction scenarios, and as such, included records of allergic reactions under various conditions. After their visit, all children received appropriate treatment. We ensured that all participants received the necessary medical attention throughout the study process. The children did not take any medication or use any anti-allergy drugs. There were no relatives in the family with peanut or drug allergies. Although some children's symptoms might have resolved spontaneously without treatment, all children included in the study received professional assessment and necessary monitoring in the hospital to ensure their safety and the accurate recording of their symptoms. This study was approved by the Ethics Committee of Jiangxi Children's Hospital (JXSETYY-YXKY–20200048), and informed consent was obtained from the families.

### Instruments and reagents

Protein immunoblotting instrument (Wuxi ConigWorth Medical Technology Co., Ltd., eZ8), enzyme-linked immunosorbent assay (ELISA) reader (Thermo Fisher Scientific, Multiskan FC), hematology analyzer (Mindray, BC-7500). Allergen-specific IgE antibody detection test kit, MEDIWISS Analytic GmbH (National Medical Products Administration registration number 20172403363), food-specific IgG antibody detection test kit, Biomerica Inc. (enzyme-linked immunosorbent assay) (National Medical Products Administration registration number 20142405705).

### Complete blood count (CBC)

A small blood sample is collected from the patient's vein or fingertip into a test tube containing an anticoagulant such as ethylenediaminetetraacetic acid (EDTA). The blood sample must be thoroughly mixed to prevent coagulation and is processed according to the instrument's requirements before analysis. An automated hematology analyzer, the Beckman Coulter DxH 500, is used for the automatic counting of blood cells, yielding counts of Lymphocytes, Neutrophils, Monocytes, Eosinophils, and Basophils, which are utilized for further analysis.

### Fecal collection and processing

Fecal samples were collected by sterile disposable applicators under hygienic conditions and immediately transferred to sterile cryovials to minimize environmental contamination. Samples were then stored at −80 °C within 2 h to preserve the integrity of the microbial DNA for subsequent molecular analysis.

### High-throughput 16S rRNA sequencing of gut microbiota

Genomic DNA was extracted from the fecal samples using the QIAamp DNA Stool Mini Kit (Qiagen) following the manufacturer's protocol. The procedure involved a series of steps including bead beating to disrupt cell walls, proteinase K treatment for cell lysis, and a series of washes and centrifugation steps to purify the DNA. The extracted DNA was then quantified using a NanoDrop 2000 spectrophotometer and assessed for quality by 1% agarose gel electrophoresis to ensure integrity and purity suitable for downstream molecular analysis. After passing the quality check, the V3–V4 variable region of the 16S rRNA gene was amplified using primers 338F (5′-ACTCCTACGGGAGGCAGCAG-3′) and 806R (5′-GGACTACHVGGGTCWTCTAAT-3′). The PCR products were quantified using 2% agarose gel electrophoresis and the QuantusTM Fluorometer (Promega, USA). Library preparation was performed using the NEXTflexTM Rapid DNA-Seq Kit (Bioo Scientific, USA). Once qualified, the sequencing was carried out using the Illumina Miseq PE300/NovaSeq PE250 sequencer.

### Taxonomic analysis

The raw sequencing data underwent quality control analysis using the fastp software, followed by sequence merging using the FLASH software. Afterward, the UPARSE software was utilized to cluster the sequences into operational taxonomic units (OTUs) with a similarity threshold of 97%, which is a common practice to define microbial species based on sequence similarity. During this process, we applied a rigorous OTU filtering criteria to ensure the quality and reliability of our microbial community analysis. Specifically, OTUs that occurred in fewer than two samples or represented less than 0.001% of the total sequence reads were removed, as these may represent sequencing errors or rare intraspecific variations that could potentially skew the analysis. Additionally, we employed the UCHIME algorithm within the UPARSE pipeline for *de novo* and reference-based chimera detection and removal, eliminating artifacts resulting from PCR amplification errors. This step is crucial to prevent the misclassification of microbial taxa. Following the OTU filtering process, the impact on our data was significant in terms of reducing noise and increasing the signal-to-noise ratio. The filtered OTU table exhibited a reduced number of low-relative abundance OTUs, which improved the clarity of microbial community structure and facilitated more robust statistical analyses. The remaining OTUs were more biologically meaningful, representing the core microbial members of the community. This meticulous filtering approach ensured that the subsequent alpha and beta diversity analyses, as well as the taxonomic classification, were based on a solid foundation of high-quality OTU data, providing a more accurate reflection of the microbial community composition and its ecological implications. The RDP classifier tool was employed for species classification annotation of each sequence, using the Silva 16S rRNA database with a matching threshold of 70%. Alpha diversity analysis, which reflects microbial community richness and diversity, was conducted using various indices such as sob, ace, chao, shannon, and simpson. Beta diversity analysis was performed to assess the similarity or dissimilarity of community structures among different samples.

### Statistical analysis

Data analysis was conducted using SPSS 22.0 software [IBM SPSS Statistics for Windows, version 22.0 (IBM Corp., Armonk, N.Y., USA)]. Normally distributed continuous variables were presented as mean ± standard deviation, and the independent samples *t*-test was used for comparisons between groups. Principal coordinate analysis (PCoA) based on the Bray-Curtis distance algorithm was performed to assess the compositional differences between the two groups at baseline. The Anosim test was used to evaluate the differences in community composition between groups. LEfSe (linear discriminant analysis effect size) analysis was employed to detect differential species relative abundance between different groups, followed by linear discriminant analysis (LDA) to estimate the effect sizes of each species relative abundance. Friedman test, a non-parametric test for correlated samples, was applied for multiple related sample comparisons. To control the false discovery rate (FDR), the Benjamini-Hochberg procedure was applied to adjust the *p*-values obtained from multiple comparisons. A significance level of FDR ≤ 0.05 was considered statistically significant.

## Results

### General clinical data

#### Clinical manifestations of peanut allergy in outpatient children

Outpatient children with symptoms of skin itching, wheals, erythema, urticaria, and gastrointestinal symptoms such as abdominal pain, nausea, diarrhea, and bloating, as well as respiratory symptoms such as coughing, wheezing, and chest tightness, were seen in the clinic (see Table 1 for detailed distribution). Patients with peanut allergy exhibited a variety of symptoms. The most common symptoms were vomiting (52%), difficulty breathing (48%), and stomach cramps (45%). Less frequently reported symptoms included indigestion (30%), diarrhea (25%), and throat swelling (20%). Serious symptoms such as a sudden drop in blood pressure, pale skin or bluish lips, syncope, and dizziness occurred in 15%, 10%, 8%, and 5% of cases, respectively. Based on their medical history, which included exposure to peanut butter, a preliminary diagnosis of peanut allergy was made. After testing for peanut-specific Immunoglobulin E (IgE) antibodies, 29 children with PA were identified and numbered B1-29, While 27 normal control children, who underwent health examinations during the same period and tested negative for peanut IgE, were numbered A1–A27.

**Table 1:** Data from 29 children with peanut allergy and Data from 27 children with Ctl children.

children with PA						children with Ctl			
Sample	Gender	Age (month)	Exposure to peanut products	Symptom manifestation.	IgE	Sample	Gender	Age (month)	IgE
1	M	19	Peanut cream	Coughing	20.4	1	M	18	Negative
2	F	20	Peanut oil (pressing)	Asthma	15.5	2	M	28	Negative
3	F	22	Peanut butter	Coughing	40.2	3	M	18	Negative
4	M	24	Biscuits	Facial swelling	35.1	4	M	20	Negative
5	F	31	Peanut butter	Asthma	20.6	5	F	33	Negative
6	F	33	Biscuits	Urticaria	40.4	6	F	30	Negative
7	F	32	Biscuits	Abdominal pain or diarrhea.	37.6	7	F	23	Negative
8	F	18	Peanut oil (pressing)	Coughing	20.6	8	F	18	Negative
9	M	18	Peanut butter	Coughing	35.1	9	F	18	Negative
10	F	20	Peanut oil (pressing)	Asthma	32.1	10	F	20	Negative
11	F	22	Peanut cream	Abdominal pain or diarrhea.	23.5	11	F	22	Negative
12	F	23	Peanut butter	Facial swelling	16.1	12	F	23	Negative
13	F	21	Peanut cream	Asthma	24.3	13	F	21	Negative
14	F	25	Peanut cream	Asthma	24.1	14	F	20	Negative
15	M	24	Peanut cream	Coughing	15.6	15	M	21	Negative
16	M	30	Biscuits	Asthma	18.9	16	M	30	Negative
17	M	32	Biscuits	Urticaria	17.6	17	F	31	Negative
18	M	33	Peanut butter	Urticaria	32.2	18	M	35	Negative
19	M	19	Peanut oil (pressing)	Abdominal pain or diarrhea.	33.1	19	M	32	Negative
20	M	20	Peanut cream	Asthma	16.7	20	F	22	Negative
21	F	22	Peanut butter	Asthma	40.1	21	F	20	Negative
22	M	25	Peanut butter	Coughing	35.6	22	M	18	Negative
23	F	26	Peanut oil (pressing)	Facial swelling	35.7	23	F	20	Negative
24	M	32	Biscuits	Urticaria	36.2	24	M	34	Negative
25	F	36	Biscuits	Urticaria	15.7	25	F	36	Negative
26	M	30	Biscuits	Asthma	15.8	26	M	32	Negative
27	M	22	Peanut butter	Coughing	16.5	27	M	20	Negative
28	M	20	Peanut butter	Coughing	27.8				
29	M	25	Biscuits	Coughing	36.5				
					26.88±9.15				Negative

#### IgE levels

The IgE glade results of the children are shown in Table 1. The IgE levels of children with PA ranged from 15.5 kU/L to 40.3 kU/L, while the IgE levels of the normal Ctl group were negative. For the blood routine test results of the samples please see Table 2.

**Table 2:** Details of blood routine examination results of peanut allergy and normal children.

Group	WBC	PLT	NEU%	NEU	LYM%	LYM	MON%	MON	EOS%	EOS	BAS%	BAS
AP	9.27 ± 2.03	399.57 ± 70.23	0.49 ± 0.10*	4.71 ± 1.90*	0.40 ± 0.08*	3.62 ± 0.6*	0.07 ± 0.01	0.61 ± 0.19	0.03 ± 0.02	0.30 ± 0.14	0.003 ± 0.005	0.04 ± 0.01
Ctl	9.20 ± 2.45	324.22 ± 87.13	0.30 ± 0.07	2.79 ± 1.14	0.58 ± 0.08	5.28 ± 1.42	0.06 ± 0.02	0.60 ± 0.22	0.057 ± 0.02	0.52 ± 0.27	0.002 ± 0.004	0.02 ± 0.01

WBC, white blood; PLT, blood platelet; NEU%, neutral cell ratio; NEU, number of neutral cells; LYM%, lymphocyte ratio; LYM, lymphocyte; MON%, monocyte ratio; MON, monocyte; EOS%, eosinophilic ratio; EOS, eosinophils; BAS%, basophilic granulocyte ratio; BAS, basophilic granulocyte.

* Significant differences compared to normal children (*P* < 0.05).

#### Significant differences in alpha and beta diversity indices in peanut allergy group

**Figure 1:**
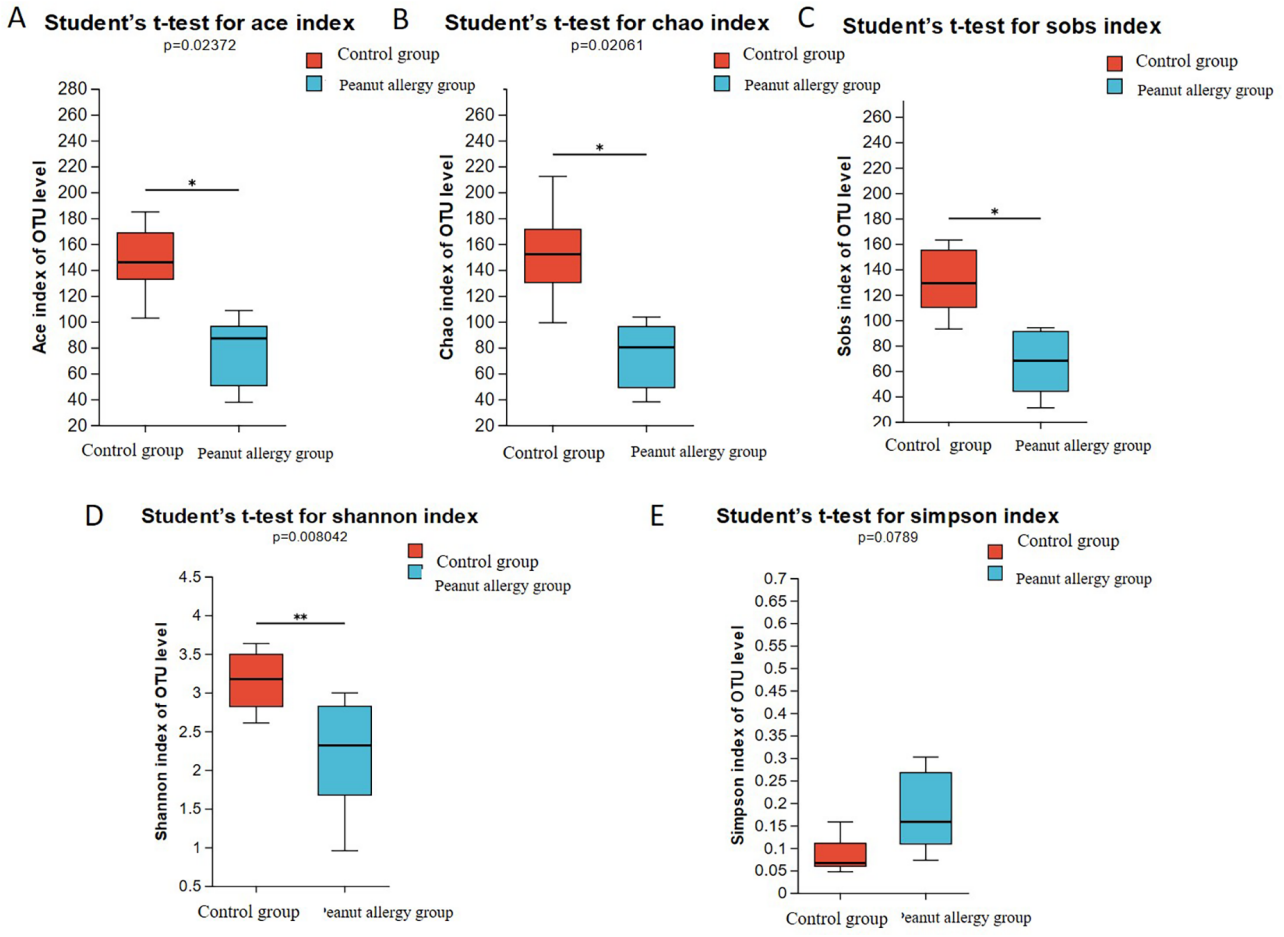
Analysis of gut microbiota alpha diversity in PA group and Ctl group. **(A)** Ace index, **(B)** Chao index, **(C)** Sobs index, **(D)** Shannon index, **(E)** Simpson index. The bar chart represents the mean values of the two groups, and the error bars represent the standard error. *Indicates FDR ≤ 0.05 compared to the control group, and **indicates FDR ≤ 0.01 compared to the control group.

The Sob index is an indicator reflecting species richness, representing the actual number of observed species in a sample. The Chao and Ace indices also reflect species richness, with the Chao index estimating the number of OTUs in a sample, and a higher value indicating a greater number of species in the sample. The Ace index evaluates both the relative abundance and evenness of species composition in a sample, with a higher value indicating greater species richness and more even distribution. In this study, all three indices (Sob, Chao, and Ace) were lower in the peanut-allergic test group compared to the control group, and the differences were statistically significant (FDR ≤ 0.05). Please refer to Figure 1A (Ace index), Figure 1B (Chao index), Figure 1C (Sob index) for visual representation. The Shannon index reflects species diversity, including both species richness and evenness of distribution among individuals. A higher Shannon value indicates higher species richness in the sample. The Simpson index also represents species diversity and measures the probability that two samples taken from a community belong to the same species. A higher Simpson index indicates lower species richness in the sample. From Figure 1D, it can be observed that the Shannon index was significantly lower in the peanut-allergic test group compared to the control group (FDR ≤ 0.01), while the Simpson index showed a higher value in the allergic group, suggesting lower species richness compared to the control group, although the difference did not reach statistical significance (Figure 1E).

**Figure 2:**
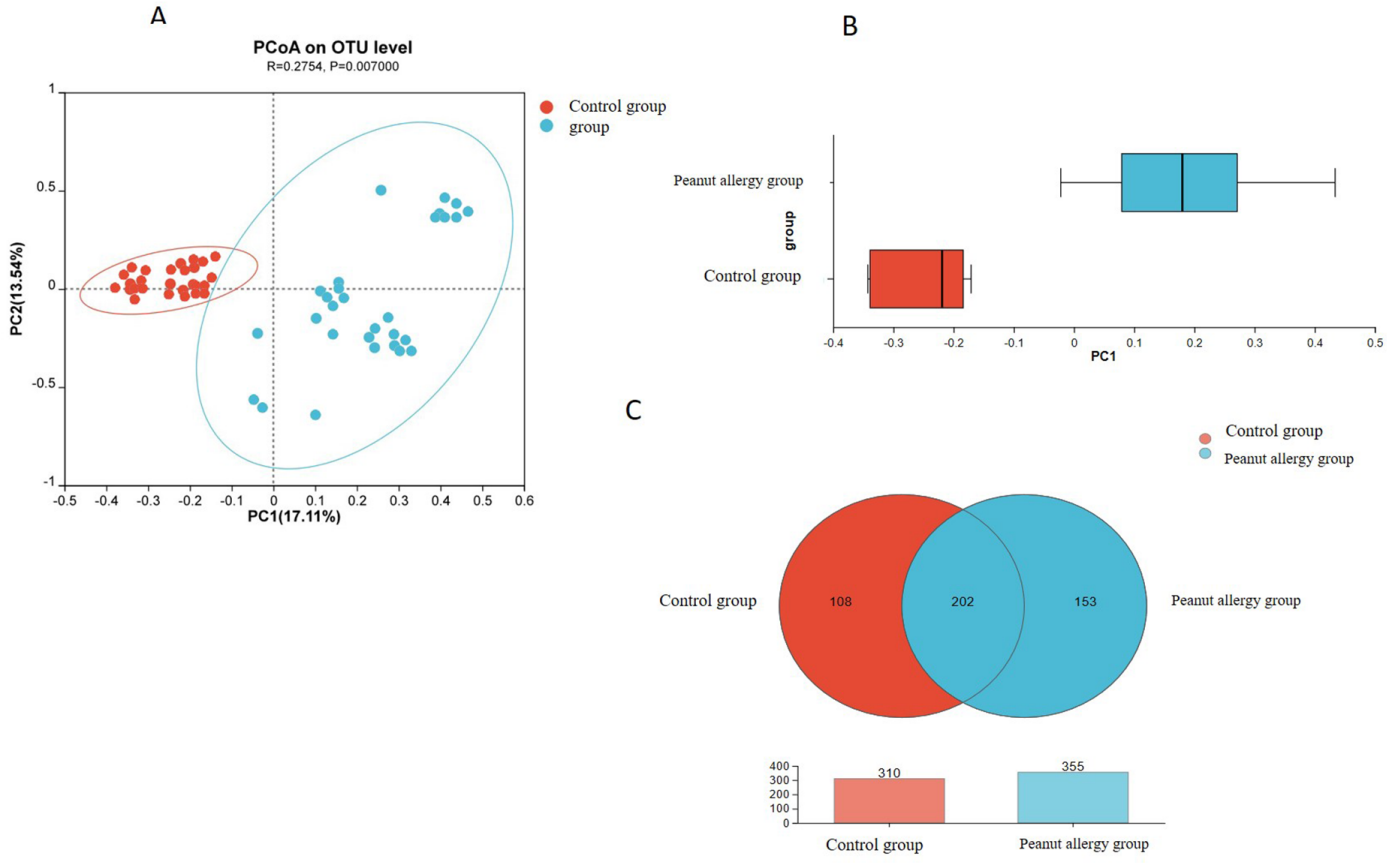
(A) Analysis of gut microbiota Beta diversity in PA group and Ctl group. **(A)** PCoA. **(B)** Presents a box plot representing the distribution dispersion of different sample groups along the PC1 axis. **(C)** Species Venn Diagram Analysis. OTUs in the control group is 310, while it is 355 in the PA group. Among these, there are 202 shared OTUs between the Ctl and PA groups, with 108 unique OTUs in the Ctl group and 153 unique OTUs in the PA group. PCoA, principal coordinate analysis; OTUs, operational taxonomic units.

In this study, we observed significant differences in Beta diversity of microbial community composition between the peanut allergy group and the control group, which are intuitively displayed in Figure 2. Figure 2A shows the PCoA, which is a non-constrained data dimension reduction analysis method. Figure 2A presents a box plot representing the distribution dispersion of different sample groups along the PC1 axis. The comparison analysis based on microbial community diversity showed certain differences in the composition of the gut microbiota between the control and peanut allergic test groups.

The Partial least squares Discriminant Analysis (PLS-DA) analysis demonstrated that the gut microbiota composition of peanut-allergic subjects was distinctly different from that of healthy individuals, forming two distinct clusters. This indicates a significant difference in gut microbiota composition between peanut-allergic subjects and healthy individuals (Figure 2B).

#### Distinct microbial community structures and key taxa associated with peanut allergy

The Venn diagram can be used to count the shared and unique species (e.g., OTUs) among multiple groups or samples, providing an intuitive representation of the similarity and overlap of species (OTUs) composition in environmental samples. Figure 2C shows that the total number of OTUs in the Ctl group is 310, while it is 355 in the PA group. Among these, there are 202 shared OTUs between the Ctl and PA groups, with 108 unique OTUs in the Ctl group and 153 unique OTUs in the PA group.

**Figure 3:**
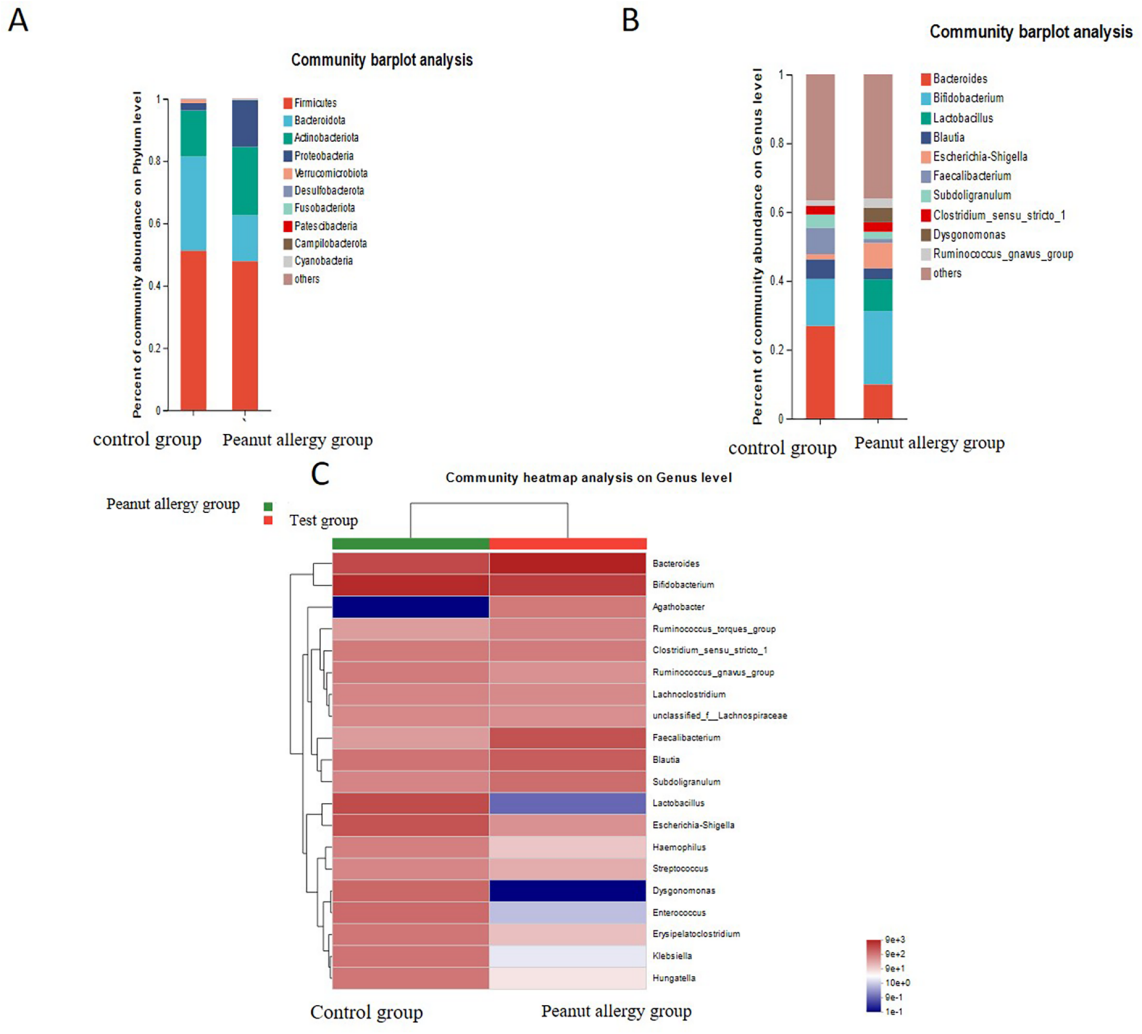
The relative relative abundance of different gut microbial taxa in the peanut allergy group compared to the control group. The bar chart represents the average relative relative abundance of each taxon in the two groups. **(A)** At the phylum level, the relative abundance of Firmicutes in peanut-allergic children decreased from 51.28% to 47.86%, while that of Bacteroidetes decreased from 30.19% to 14.74%, Actinobacteria increased from 14.82% to 21.99%, and Proteobacteria increased significantly from 2.32% to 14.95%. **(B)** Bacteroides, Blautia, and Faecalibacterium were significantly decreased in allergic children, while Bifidobacterium, Lactobacillus, Escherichia-Shigella, and Dysgonomonas were significantly increased. **(C)** The heatmap of species richness at the genus level also showed significant differences between the Ctl and PA groups.

Further analysis revealed that, as shown in Figure 3A at the phylum level, compared with the Ctl group, the relative abundance of Firmicutes in peanut-allergic children decreased from 51.28% to 47.86%, while that of Bacteroidetes decreased from 30.19% to 14.74%, Actinobacteria increased from 14.82% to 21.99%, and Proteobacteria increased significantly from 2.32% to 14.95%. At the genus level (Figure 3B), it was observed that Bacteroides, Blautia, and Faecalibacterium were significantly decreased in PA children, while Bifidobacterium, Lactobacillus, Escherichia-Shigella, and Dysgonomonas were significantly increased. The heatmap of species richness at the genus level also showed significant differences between the Ctl and PA groups (Figure 3C).

#### Comparison of gut microbiota composition differences between peanut allergic group and control group

**Figure 4:**
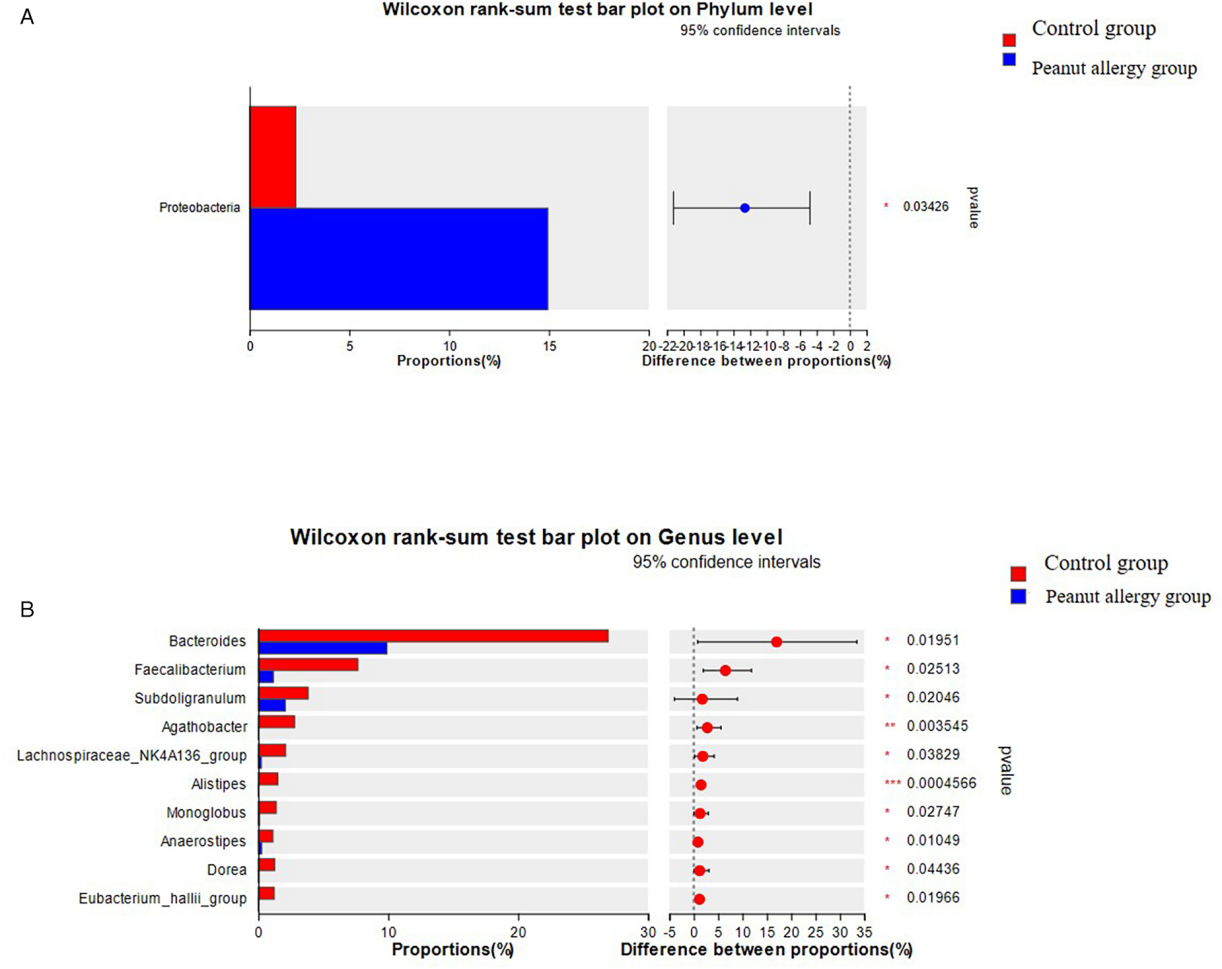
Intergroup differences in gut microbiota composition between the PA group and Ctl group. **(A)** Proteobacteria in the gut microbiota of peanut-allergic children compared to healthy children, and this difference was statistically significant. **(B)** Relative abundance of Bacteroides, Faecalibacterium, Subdoligranulum, Agathobacter, Lachnospiraceae_NK4A136_group, Alistipes, Monoglobus, Anaerostipes, Dorea, and Eubacterium_hallii_group was significantly lower in PA children compared to Ctl children (FDR ≤0.05 or FDR ≤0.01).

At the phylum level, there was a significant increase in Proteobacteria in the gut microbiota of PA children compared to Ctl children, and this difference was statistically significant (FDR ≤ 0.05) (Figure 4A). At the genus level, the relative abundance of Bacteroides, Faecalibacterium, Subdoligranulum, Agathobacter, Lachnospiraceae_NK4A136_group, Alistipes, Monoglobus, Anaerostipes, Dorea, and Eubacterium_hallii_group was significantly lower in PA children compared to Ctl children (FDR ≤ 0.05 or FDR ≤ 0.01) (Figure 4B).

#### Lefse multi-level species discrimination analysis

**Figure 5:**
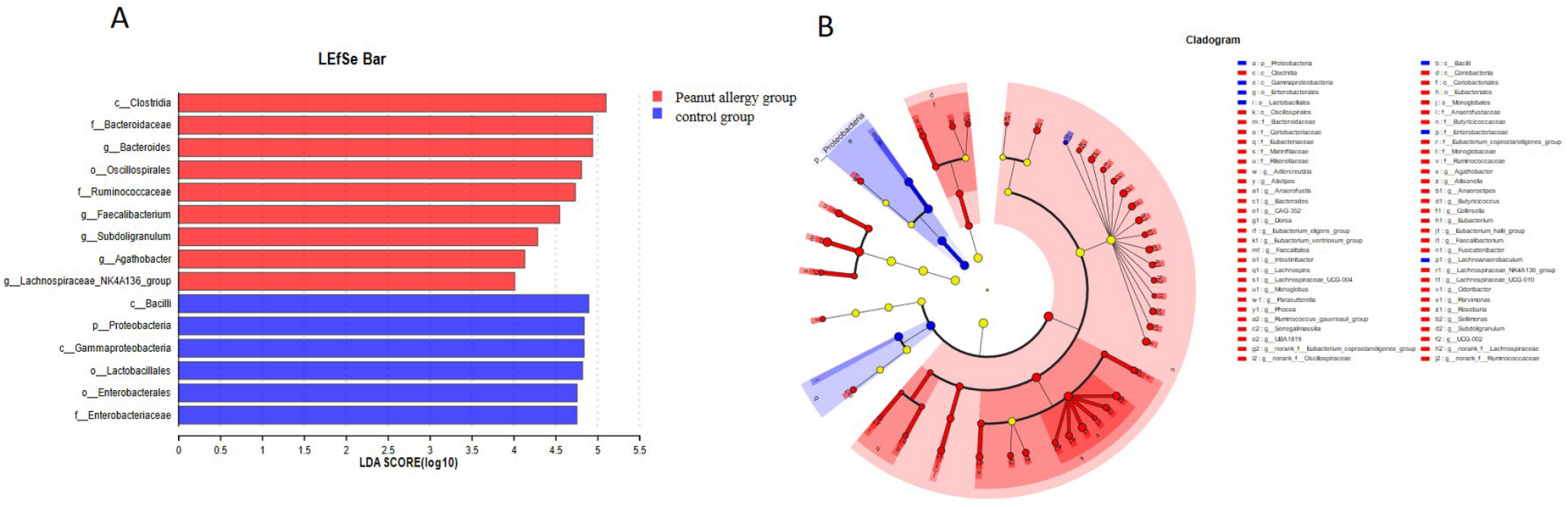
Lefse multi-level species difference discriminant analysis. **(A)** Microbial taxa significantly associated with peanut allergy, including Bacilli, Proteobacteria, Gammaproteobacteria, Lactobacillales, Enterobacterales, and Enterobacteriaceae. Red bars: Represent the relative relative abundance or level of significance of certain microbial groups in the gut microbiota of children in the normal control group. Blue bars: Represent the relative relative abundance or level of significance of the corresponding microbial groups in the test group (peanut allergy, AP) children. In the multi-level species differential analysis (LEfSe), the height of the bars may indicate the Linear Discriminant Analysis (LDA) score, which is a statistical method for measuring the impact of categorical variables. The higher the LDA score, the stronger the discriminative ability of the microbial group in distinguishing between the two groups (such as the peanut allergy group and the healthy control group). **(B)** Illustrates the microbial communities that exhibit significant differences between the peanut allergy group and the healthy control group across various taxonomic levels, such as phylum, class, order, family, and genus.

Lefse analysis allows for differential testing at multiple taxonomic levels, including phylum, class, order, family, genus, and species. It identifies differentially abundant taxa and uses LDA values to measure the effect sizes of each taxon on the observed differences, indicating their potential key roles in disease development. Figure 5A shows the microbial taxa significantly associated with peanut allergy, including Bacilli, Proteobacteria, Gammaproteobacteria, Lactobacillales, Enterobacterales, and Enterobacteriaceae. Figure 5B displays the differentially abundant taxa at multiple taxonomic levels.

### Strong correlations between IgE levels and specific microbial groups in peanut allergy

**Figure 6:**
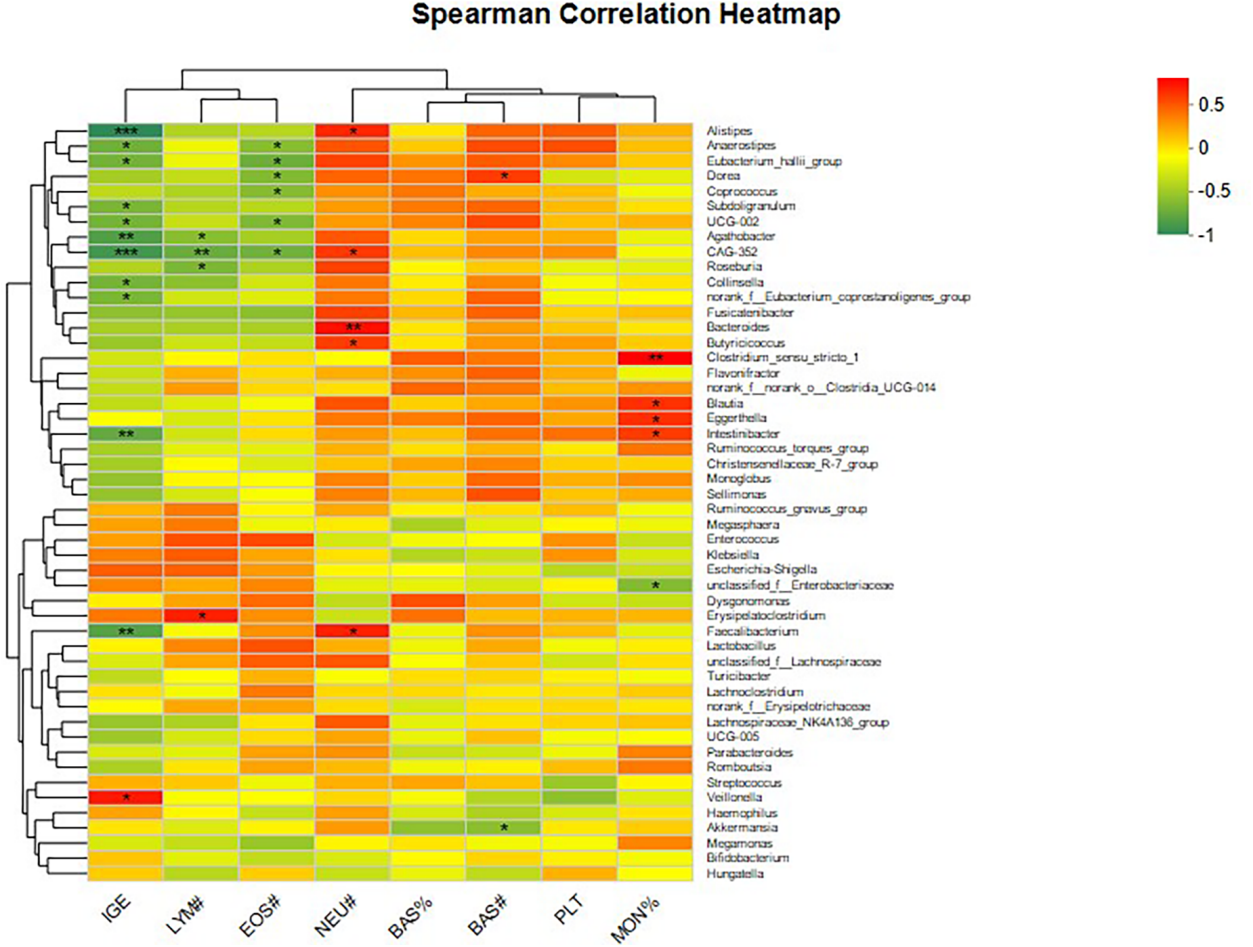
Heatmap of the correlation between the top 50 advantageous microorganisms and clinical factors. *Will be used to denote the levels of statistical significance. *Indicates FDR ≤ 0.05, **indicates FDR ≤ 0.01, and ***indicates FDR ≤ 0.001. Colors will represent the strength of the correlation, with varying shades or saturation levels to distinguish from weak to strong correlations. For instance, a light blue may signify a weaker correlation, while a darker shade of blue indicates a stronger correlation.

By examining the correlation coefficients between the top 50 dominant microbes and clinical factors, we can identify which microbes have a strong correlation with clinical factors/disease phenotypes. These microbes may be key species that affect clinical factors/disease phenotypes and can be used as one of the ways to screen microbial biomarkers for disease and healthy groups (Figure 6).

#### Correlation of IgE levels with microbiota

We observed a negative correlation between IgE levels and the microbiota Alistipes and CAG-352 (FDR < 0.001), Agathobacter and Intestinibacter (FDR < 0.01), Anaerostipes (FDR < 0.05), Eubacterium_hallii_group (FDR < 0.05), Subdoligranulum (FDR < 0.05), UCG-002 (FDR < 0.05), and Collinsella (FDR < 0.05). These results suggest that these microbial communities may be key factors in regulating IgE levels, thereby influencing the response to peanut allergy.

#### Correlation of hematological parameters with microbiota

In hematological parameters, the neutrophil (NEU), basophil (BAS), and monocyte percentage (MON%) showed a positive correlation with the gut microbiota, while the eosinophil (EOS) showed a negative correlation. These findings may help us understand how the gut microbiota affects the immune system's response to peanut allergens.

#### Indicators without statistical significance

Basophil percentage and platelet count show no statistical significance in correlation with various species of bacteria.

#### Indicators with strong and significant correlation

IGE has a strong and significant correlation (FDR ≤ 0.01) with Alistipes, Agathobacter, CAG-352, Intestinibacter, and Clostridium. The neutrophil count has a strong and significant correlation (FDR ≤ 0.01) with Bacteroides. Monocyte percentage has a strong and significant correlation (FDR ≤ 0.01) with Clostridium_*sensu*_*stricto*_1.

In PA children, except for Veillonella, Alistipes and CAG-352 (FDR ≤ 0.001), Agathobacter and Intestinibacter (FDR ≤ 0.01), Anaerostipes (FDR ≤ 0.05), Eubacterium_hallii_group (FDR ≤ 0.05), Subdoligranulum (FDR ≤ 0.05), UCG-002 (FDR ≤ 0.05), and Collinsella (FDR ≤ 0.05) were negatively correlated with blood IgE. In addition, NEU, BAS, and MON% in the blood routine index were positively correlated with gut microbiota, while EOS was negatively correlated with gut microbiota.

In summary, our results reveal significant differences in gut microbial community diversity and composition between children with peanut allergy and healthy control subjects, which may have important implications for understanding the pathogenesis of peanut allergy.

## Discussion

Our main finding is that the relative relative abundance of the Proteobacteria phylum in the gut microbiota of children with peanut allergy significantly increased, while the relative relative abundance of the Firmicutes and Bacteroidetes phyla significantly decreased, a result that is intuitively displayed in Figure 3. Specifically, compared to the healthy control group, the relative relative abundance of the Proteobacteria phylum in the peanut allergy group increased from 2.32% to 14.95% (FDR ≤ 0.05), while the relative relative abundance of the Firmicutes and Bacteroidetes phyla decreased from 51.28% to 47.86% and from 30.19% to 14.74%, respectively (FDR ≤ 0.05). These changes suggest that peanut allergy may be associated with significant alterations in the structure of the gut microbiota. We found that children with peanut allergy had reduced gut microbiota diversity, particularly a decrease in Firmicutes and Bacteroidetes, and an increase in Proteobacteria. These changes may be related to the abnormal immune regulation in children with peanut allergy, where the increase in Proteobacteria may be associated with exacerbated inflammatory responses (24).

Additionally, we observed that the relative abundance changes of specific bacterial genera in children with peanut allergy are negatively correlated with the increase in serum IgE levels, which further supports the notion that the gut microbiota may be involved in the development of peanut allergy by modulating immune responses (25). For instance, the relative abundance of Alistipes and CAG-352 is associated with a decrease in IgE levels, while the relative abundance of Veillonella is associated with an increase in IgE levels, which may indicate the roles these microbes play in peanut allergy.

These findings not only provide a new perspective for understanding the microbiome basis of peanut allergy, but also offer potential targets for the development of new prevention and treatment strategies. Future research needs to further explore the causal relationship between the gut microbiota and peanut allergy, and assess the potential effectiveness of modulating the gut microbiota as a treatment for peanut allergy.

Food allergy (FA) is defined as “an adverse health effect arising from a specific immune response that occurs reproducibly on exposure to a given food” (26). In recent years, increasing research has shown a close relationship between gut microbiota and various food allergies (27–30). Our findings indicate a robust correlation between alterations in gut microbiota and IgE levels in children with peanut allergy. Specifically, the IgE levels exhibited a strong negative correlation with the relative abundance of certain microbial groups, such as Alistipes and CAG-352 (FDR ≤ 0.001), and a positive correlation with Veillonella. This association suggests that the gut microbiota may play a pivotal role in the immunopathogenesis of peanut allergy by modulating the production of IgE antibodies, which are hallmarks of type I hypersensitivity reactions (31). The observed correlations are in line with emerging evidence that the gut microbiome is a critical regulator of immune responses, including those involved in food allergies. The potential mechanisms could involve the production of metabolites that influence immune cell activity, the regulation of the intestinal barrier function, and the direct interaction with immune cells to shape the immune response. IgE levels were strongly correlated with the relative abundance of Parabacteroides, Bacteroides, CAG-352, Enterobacteriaceae, and Clostridium_*sensu*_*stricto*_1, while neutrophil percentage was strongly correlated with the relative abundance of Prevotella. Monocyte percentage was strongly correlated with Clostridium_*sensu*_*stricto*_1 and had significant significance. Taking corresponding medications to improve the composition of the microbiota may be a possible way to restore normal microbiota. The strong correlation between neutrophils, monocytes, and bacteria can assist in clinical diagnosis and treatment.

Comparing our results with those from other studies, we found both consistencies and discrepancies, which may reflect differences in study populations, methodologies, or environmental factors. For instance, while some studies have also reported a decrease in beneficial bacteria such as Bacteroidetes and Firmicutes in allergic individuals, others have noted an increase in Proteobacteria. The clinical implications of our findings are profound. Understanding the gut microbiota's influence on IgE levels could lead to the development of microbiota-targeted therapies, such as probiotics or prebiotics, to prevent or treat peanut allergy. Moreover, the identified microbial groups could serve as potential biomarkers for the diagnosis and monitoring of peanut allergy. Given the complexity of the gut microbiome and its interaction with the host immune system, further research is warranted. Future studies should aim to elucidate the causal relationships between specific microbial changes and the development of peanut allergy. Additionally, interventional studies could explore the efficacy of modulating the gut microbiota as a therapeutic strategy for food allergies.

Comparing our results with those from similar studies, we observed both similarities (32) and differences, which may be attributed to variations in study design, population characteristics, and environmental factors. The observed diversity patterns in our study may also be influenced by factors such as diet, antibiotic use, and early-life exposures, which are known to shape the gut microbiota. The implications of reduced microbial diversity for health are multifaceted. A less diverse gut microbiota may lead to a diminished capacity for immune system development and a reduced ability to prevent or mitigate allergic reactions. This suggests that interventions aimed at enhancing gut microbiota diversity could be a promising avenue for allergy prevention and treatment. Given the importance of gut microbiota diversity for health, future research should explore specific interventions that can promote a more diverse gut ecosystem, such as dietary modifications, probiotics, and prebiotics. Additionally, longitudinal studies are needed to understand how early-life events impact the development of gut microbiota diversity and its relationship with the risk of developing allergies.

The comparative analysis of gut microbiota composition between children with peanut allergy and the control group revealed notable differences at various taxonomic levels. Particularly, the phylum Firmicutes and Bacteroidetes were found to be less abundant in the peanut allergy group, while the Proteobacteria phylum was significantly increased. These shifts in microbial composition may have profound implications for immune system development and function. The decrease in Firmicutes, known for their role in the production of short-chain fatty acids that modulate immune responses, could contribute to a less regulated immune system, potentially leading to allergic reactions. Conversely, the increase in Proteobacteria, which includes various pathogenic species, might be indicative of a pro-inflammatory state that could exacerbate allergic sensitivities. Recent studies have found that Proteobacteria may play an important role in peanut allergies. Proteobacteria is a type of bacteria widely present in nature, including many pathogenic bacteria in the human body and environment. In the gut microbiota of food allergy patients, the proportion of Proteobacteria significantly increases, which is consistent with our research results (33–35). Another change in the microbiota from the phylum Firmicutes, which comes from other groups of bacteria in the phylum Firmicutes, is observed in infants who are breastfed. In the early stages, infants who are breastfed show a dominance of bacteria from the phylum Firmicutes, such as Bifidobacterium, Lactobacillus, and Veillonella. However, with the introduction of solid foods, this dominance shifts to bacteria from the phylum Bacteroidetes and Clostridiales (8, 36). The microbial composition stabilizes around the ages of 3–4 and remains consistent into adulthood (37). The first few years of life are a vulnerable period for the development of the microbiota, and events such as infections and fevers can directly or indirectly impact the microbiota. Some of these changes may persist over time. The early-life microbiota plays an important role in the development of tolerance to immune function and in preventing adverse inflammatory reactions against self-antigens and non-self-antigens (e.g., allergens). Changes in the relative abundance of bacteria from the phylum Firmicutes, such as Veillonella, are significantly associated with allergies (38). Our study found a decrease in the proportion of bacteria from the phylum Firmicutes in the gut microbiota of infants with peanut allergies, from 51.28% to 47.86%. Bacteroidota may also play a certain role in peanut allergies. Bacteroidota is a common gut microbiota, and its changes in species and quantity are closely related to gut health (39). After the introduction of complementary foods, the relative abundance of Bacteroides increases in infants and young children. However, we have found a significant decrease in the types and quantities of this phylum in children with peanut allergies, which is closely related to gut health. The relative abundance of Bacteroidetes decreased from 30.19% to 14.74%. A study through mouse experiment found that feeding mice with strains containing Bacteroides fragilis could significantly alleviate peanut allergies, indicating that Bacteroidota may play an important role in peanut allergies (40). Bacteroidota can participate in the regulation of various metabolic and immune functions in the human body (41). One other study found that the proportion of Bacteroidota is generally lower in food allergy patients (27). In peanut allergy patients, the level of Bacteroidota is usually even lower. In addition, the study also found that increasing the intake of probiotics Lactobacillus rhamnosus and Bifidobacterium longum can promote the growth of Bacteroidota and alleviate peanut allergy symptoms. Among the genus level of gut microbiota in children with peanut allergies, Bacteroides, faecalibacterium, subdoligranulum (42), Alistipes, monoglobus, anaerostipes, dorea, agathobacter, lachnospiraceae_NK4A136_group, and eubacterium_hallii_group are significantly lower than in normal children. Among them, faecalibacterium is an anaerobic bacterium that does not produce spores and is a non-motile Gram-positive rod. It can produce a large amount of butyrate, formate, and a small amount of D-lactic acid after fermenting glucose, and has the function of reducing inflammation and improving the intestinal barrier. The results showed that children with peanut allergies had significantly reduced diversity and species diversity of intestinal microbiota, and there were significant differences in microbial clustering between the two groups. The relative relative abundance of Enterobacteriaceae and Bacteroides increased in the peanut allergy group, while the relative relative abundance of Prevotella decreased, indicating a close relationship between peanut allergy and changes in intestinal microbiota.

Our findings are in line with some studies that have reported similar changes in microbial composition in the context of other allergic conditions. However, discrepancies with other research highlight the complexity of the relationship between gut microbiota and allergic diseases, which may be influenced by a multitude of factors, including genetic predisposition, environmental exposures, and lifestyle choices. From an ecological perspective, the observed changes in gut microbiota could reflect an altered ecosystem stability and functionality, which may impact the host's ability to maintain homeostasis. This could involve alterations in the production of essential metabolites, changes in the intestinal barrier integrity, and modulation of immune cell development and activity. Environmental factors, such as diet, which is known to be a major driver of gut microbiota composition, could partially explain the observed differences. Future research should investigate how dietary interventions might be leveraged to modulate the gut microbiota and potentially mitigate allergic symptoms. The interaction between the host's immune system and the gut microbiota is bidirectional. Further studies should explore the mechanisms by which specific microbial changes influence the host's immune response to allergens, and vice versa.

Further research found that the microbial groups that significantly contribute to peanut allergies are Bacilli, Proteobacteria, Gammaproteobacteria, Lactobacillales, Enterobacterales, and Enterobacteriaceae. Gu et al. found that the gut microbiota composition was reshaped in peanut-allergic mice using a peanut allergy model in Balb/c mice by 16S rRNA sequencing and analyzed the correlation between allergic indicators and gut microbiota composition. Outcomes showed that the gut microbiota composition was reshaped in peanut-allergic mice, with Acidobacteriota, Lachnospiraceae, Rikenellaceae, Alistipes, Lachnospiraceae_NK4A136_group significantly down-regulated and Muribaculaceae up-regulated (40).

The findings of our study carry significant implications for the clinical management of peanut allergy. The observed differences in gut microbiota composition between children with peanut allergy and the control group suggest potential avenues for diagnostic and therapeutic interventions. As the gut microbiota is modifiable, our results support the exploration of targeted interventions to restore a balanced microbial ecosystem. Probiotics, prebiotics, and dietary adjustments could be considered as complementary approaches to traditional allergy management. The potential use of certain microbial groups as diagnostic biomarkers for peanut allergy warrants further investigation. Identifying such markers could lead to earlier and more accurate diagnoses, as well as personalized treatment plans. Our findings also highlight the need for preventive strategies to modulate the gut microbiota in early life, potentially reducing the risk of developing peanut allergies. This could involve promoting a diverse diet and avoiding unnecessary antibiotic use in infants and young children. Personalized medicine, tailored to an individual's gut microbiota profile, may offer a more effective approach to managing peanut allergy. By understanding a patient's unique microbial composition, clinicians can provide tailored advice and treatment options. Longitudinal studies are necessary to track the long-term impact of gut microbiota modulation on the course of peanut allergy. This will provide insights into the sustainability of interventions and their effects on disease progression. Educating patients and their families about peanut allergy is crucial. This includes awareness of allergen avoidance, symptom recognition, and the potential role of gut health in allergy management.

While our study provides valuable insights into the relationship between gut microbiota and peanut allergy, it is not without limitations. Firstly, the sample size, though adequate for preliminary analysis, may not fully represent the broader population. This limits the generalizability of our findings and calls for larger, multicenter studies to confirm our results. Secondly, our cross-sectional design does not allow for causal inferences. Future research should employ longitudinal or interventional study designs to better understand the temporal relationships and causal directions between gut microbiota changes and the development of peanut allergy. The mechanisms by which specific microbial changes influence the immune response in peanut allergy remain unclear and warrant further investigation. Future studies should incorporate advanced molecular and immunological techniques to elucidate these mechanisms. Intervention studies are needed to test the efficacy of modulating the gut microbiota as a therapeutic strategy. This could involve clinical trials of probiotics, prebiotics, or dietary interventions aimed at enhancing the diversity and function of the gut microbiota in children at risk of or with peanut allergy. Our study also highlights the need for interdisciplinary research that combines expertise from microbiology, immunology, nutrition, and pediatrics to advance our understanding of the complex interactions between diet, gut microbiota, and allergic diseases. Longitudinal studies are essential to assess the long-term impact of gut microbiota modulation on the natural history of peanut allergy and to evaluate the sustainability of interventions over time.

## Conclusion

The relative abundance of the Proteobacteria phylum in the gut microbiota of children with peanut allergy has significantly increased, while the relative abundance of the Firmicutes and Bacteroidetes phyla has significantly decreased, which may be associated with the abnormal immune regulation in children with peanut allergy.

The reduction in gut microbiota diversity in children with peanut allergy, particularly the decrease in Firmicutes and Bacteroidetes, along with the increase in Proteobacteria, may be related to the pathogenesis of peanut allergy.

Changes in the relative abundance of specific bacterial genera in children with peanut allergy are negatively correlated with changes in serum IgE levels, indicating that the gut microbiota may be involved in the development of peanut allergy by modulating immune responses.

The results of this study support the possibility of the gut microbiota as a potential therapeutic target for peanut allergy and suggest that future research should further explore the modulation of the gut microbiota as a strategy for treating peanut allergy.

## Data Availability

The raw data supporting the conclusions of this article will be made available by the authors, without undue reservation.
